# Polymicrobial urine cultures: reconciling contamination with the urobiome while recognizing the pathogens

**DOI:** 10.3389/fcimb.2025.1562687

**Published:** 2025-05-19

**Authors:** Robert B. Moreland, Linda Brubaker, Alan J. Wolfe

**Affiliations:** ^1^ Department of Microbiology and Immunology, Loyola University Chicago, Maywood, IL, United States; ^2^ Department of Obstetrics, Gynecology and Reproductive Sciences, University of California, San Diego, La Jolla, CA, United States

**Keywords:** contamination, mixed culture, polymicrobial, urine culture, urinary tract infection, urogynecology, urology

## Abstract

Polymicrobial or mixed urine cultures of more than one predominant microbe confound clinical urinary tract infection diagnosis. The current College of American Pathologists clinical laboratory standard states that a urine sample cultured with more than two isolates with >10,000 colony forming units/ml is to be considered contaminated. However, the presence of urinary sample bacteria in individuals without urinary symptoms (referred to as asymptomatic bacteriuria) is common especially in older people and in pregnant individuals. Furthermore, the discovery of an indigenous urinary microbiome (urobiome) in healthy humans throughout life from shortly after birth to death conflicts with the long-standing notion that urine derived from sterile filtered blood should be sterile above the urethral sphincter. Polymicrobial infections are not consistent with Koch’s postulates that a single pathogen is causal for disease. In this review, we will discuss current standards of contamination, how to reconcile the sterility of urine with the existence of the urobiome, a history of polymicrobial infections, and why re-examining current practices is essential for the practice of medicine, improving quality of life, and potentially saving lives.

## 1 Introduction

The College of American Pathologists guidance states “a contaminated urine culture was defined as the presence of more than 2 isolates at greater than or equal to 10–000 CFU/mL” (Valenstein and Meier, 1998; Bekeris et al., 2008). This includes commensal microbes thought to be of skin origin or, in adult females, vulvo-vaginal contaminants (Brubaker et al., 2021a). Standard practice is to resample although often the same result is obtained. Then, the predominant microbe is reported (often *E. coli*), while the other detected microbes are often ignored (Sfeir and Hooton, 2018). Examples include reports of “contamination” or “mixed flora” in asymptomatic bacteriuria of pregnant individuals (46.7%) (O'Leary et al., 2020), in general practice (54.9%) (Hansen et al., 2022), and in outpatient clinics (46.2%) (Whelan et al., 2022). This is comparable to results obtained with a multiplex polymerase chain reaction (M-PCR) panel, where 56.1% of an older cohort (≥65 years old) with diagnosed urinary tract infection (UTI) had polymicrobial (more than one microbe) results (Vollstedt et al., 2020).

While the method of urine collection can sample different aspects of the urinary tract (e.g., suprapubic aspirate and transurethral catheter: bladder and upper urinary tract; midstream void: entire urinary tract including urethra and peri-urethral skin plus urogenital regions), there are subtle differences in microbes detected depending on sampling technique (Chen et al., 2020; Pohl et al., 2020; Brubaker et al., 2021a; Wang et al., 2023; Du et al., 2024; Shafik et al., 2005; McFadyen and Eykyn, 1968; Wolfe et al., 2012) **Table 1**. Nevertheless, midstream void or the so-called “clean catch” is most often used in clinical practice (LaRocco et al., 2015; Moreland et al., 2024). A recent meta-analysis of urine collection methods and contamination concluded that methods to decrease contamination (e.g. cleansing, boric acid preservative, and refrigerating urine sample to prevent nonspecific growth) were of limited value (LaRocco et al., 2015). The possibility remains that many microbes reported as contamination or mixed flora could represent potential polymicrobial infection that, in some cases, may breach the renal urine blood barrier and progress to urosepsis (Siegman-Igra, 1994; Siegman-IgraY, 1994; Peach et al., 2016; Akhtar et al., 2021; Collaborators, GBD 2021 Antimicrobial Resistance, 2024). Distinguishing contamination from the presence of clinically relevant microbes is essential to test interpretation and reporting. It is helpful to understand the history of urine culture contamination.

**Table 1:** Methods of urine collection for clinical laboratory analysis.

Method	Regions sampled	Advantages	Disadvantages	References
Midstream Void Clean Catch	Kidney, Ureter, Bladder, Urethra (Prostate), Urethral Meatus, Urogenital Regions	Least invasive	Includes urethral and urogenital flora, risk of “contamination”	(Brubaker et al., 2021b; Pohl et al., 2020; Wang et al., 2023)
Transurethal Catheterization	Kidney, Ureter, Bladder	Bladder and upper urinary tract only. Excludes urethral and urogenital flora	Invasive. Catheterization	(Pohl et al., 2020; Wang et al., 2023; Du et al., 2024; Wolfe et al., 2012)
Suprapubic Aspirate	Kidney, Ureter, Bladder	Bladder and upper urinary tract only. Excludes urethral and urogenital flora	Invasive (Suprapubic needle into the bladder)	(McFadyen and Eykyn, 1968; Wolfe et al., 2012)
Ureteroscopy	Kidney, Ureter	Renal and ureteral flora only	Invasive, rarely used except under certain circumstances	(Shafik et al., 2005)

## 2 Origins of urine sample contamination

The belief that urine is sterile above the urethral sphincter is attributed to Pasteur and his studies proposing germ theory in the 1860s (Asscher et al., 1966; Roll-Hansen, 1979; Brubaker et al., 2023). The seminal fact omitted in many contemporary accounts is that Pasteur boiled the urine, vacuum sealed the flasks and observed no growth. Indeed, Pasteur considered urine alone to be an excellent bacterial growth media (Asscher et al., 1966). The point of his famous experiment was to demonstrate that the growth he observed in urine open to the environment resulted from microbes and not spontaneous generation from miasma (Roll-Hansen, 1979). Later publications by Roberts reported no detectable microbes in fresh urine of healthy subjects using the techniques of the day (e.g., microscopy) (Roberts, 1881). However, all samples left open to the air and at room temperature developed cloudiness and an ammonia odor in two to three days. This was attributed to microbes that could metabolize urea (Roberts, 1881) and later shown to be due to urease expressing facultative anaerobes such as *Proteus* (Armbruster et al., 2017).

The success of Koch’s postulates in identifying single microbes as causes of mortal diseases led to a quantum advance in the diagnosis of infectious disease in the late nineteenth century (Blevins and Bronze, 2010). As culture methods evolved for the detection of UTI-associated pathogens, protocols that detected the most common pathogen, *Bacterium coli commune* (later *Escherichia coli*), became standard clinical laboratory practice (Friedmann, 2014). Although multiple publications during the twentieth century reported microbes in the “sterile” urine of healthy individuals without symptoms (Hort, 1914; Marple, 1941; Philpot, 1956; McFadyen and Eykyn, 1968; Maskell, 1986; Maskell, 1988), special methods were necessary to culture these presumably “uncultivatable” microbes (Maskell, 1988; Khasriya et al., 2013; Hilt et al., 2014; Price et al., 2016; Legaria et al., 2022), and the dogma remained that urine was sterile in the absence of clinical conditions, such as UTI. Thus, contamination as it is currently defined is thought to arise from extravesicular (outside the bladder) sources and be unrelated to the cause of symptoms or infection; contamination is an artifact of urine sample collection (Valenstein and Meier, 1998; Bekeris et al., 2008; LaRocco et al., 2015). The prevailing view is that urine specimens can easily become contaminated with periurethral, epidermal, perianal, and vaginal flora (LaRocco et al., 2015). One report defined contamination as “bacteria that are found in normal vaginal or skin flora and do not cause UTI” (Blake and Doherty, 2006).

Urine culture contaminants attributed to dermal origin (skin) are usually identified as Gram-positive diphtheroids (*Corynebacteria*, club-like), Gram-positive clustered cocci (*Staphylococcus*), and Gram-positive cocci (*Streptococcus, Micrococcus*). A survey of the human skin microbiome identifying the ten most abundant taxa in four different regions of skin (dry, moist, sebaceous, and feet) found 22 different microbes. Of these, 21 are Gram-positive aerobes, microaerophiles or facultative anaerobes (Byrd et al., 2018) (**Supplementary Table 1**). Three are within the Viridians group streptococci (VGS) (Doern and Burnham, 2010). Only one is Gram-negative; the anaerobic coccus *Veillonella parvula* (found only in the dry region sample). Thus, skin contamination would be consistent with Gram-positive commensals.

The vaginal microbiome changes with age and reproductive status (menarche, reproductive age and post-menopausal) (Ravel et al., 2011; Nunn and Forney, 2016; Saraf et al., 2021; Park et al., 2023). Consequently, vaginal microbiota can vary with the patient. In general, the healthy vaginal microbiome is predominated by *Lactobacilli*, although other taxa have been observed (Ravel et al., 2011; Nunn and Forney, 2016; Saraf et al., 2021) (**Supplementary Table 2**). Vaginal contaminants are usually identified as Gram-positive bacilli and are attributed to commensal flora. These include *L. iners*, *L. crispatus*, *L. gasseri* and *L. jensenii* but can also include *Gardnerella vaginalis* (Gram-variable) and Gram-positive anaerobe *Atopobium vaginae* (now known as *Fannyhessea vaginae*) (Nouioui et al., 2018).

The vulvar microbiome has recently been investigated and includes representative taxa from both skin and vagina, including the genera *Corynebacterium*, *Lactobacillus*, *Staphylococcu*s, *Prevotella*, *Propionibacterium (Cutibacterium)* and *Finegoldia* (Pagan et al., 2021). These microbes are Gram-positive diphtheroids, rods, and cocci except for the Gram-negative anaerobe *Prevotella*. Assessing gastrointestinal contamination including perianal and perineal regions becomes problematic as these microbes are facultative anaerobes that have also been identified as uropathogens. These include the genera *Escherichia*, *Pseudomonas*, *Klebsiella*, *Proteus*, and Gram positive *Enterococcus* commonly reported in UTI and are the microbes most often detected using current diagnostic techniques (urine dipstick and SUC) (Flores-Mireles et al., 2015; Moreland et al., 2023) **Tables 2** and **3**. Consequently, they are diagnosed as uropathogens rather than perineal contaminants.

**Table 2:** Assays for urinary tract infection diagnosis.

Method	Basis	Detection	Advantages	Disadvantages	References
Urine Dipstick	Detects nitrite (bacteria), leukocyte esterase (infection, WBC) and pH	Colorimetric, Time to result, minutes.	Quick, accessible, Rapid diagnosis as a first read of UTI	False negatives, false positives, half of known and emerging uropathogens do not make nitrite.	(Kavuru et al., 2020; Mundt and Shanahan, 2010; Moreland et al., 2024)
Standard Urine Culture (SUC)	Plating urine on MacConkey and sheep blood agar plates, incubate aerobically 30-37C.	Bacterial plates, 12-36h,	Current clinical Gold standard.	Aerobic culture misses up to 70% of known and emerging uropathogens, negative cultures, Favors the rapid growth of facultative anaerobes	(Gillespie et al., 1960; Brubaker et al., 2023; Price et al., 2017; Moreland et al., 2024)
Expanded Quantitative Urine Culture (EQUC)	Variety of plates and atmospheric conditions	Bacterial plates, 24-48h	Detects 70% of normal urinary bladder flora, distinguishes live from dead microbes	Time to result, great research tool	(Price et al., 2016; Deen et al., 2023; Du et al., 2024)
16S rRNA/18S rDNA Gene amplification/sequencing	PCR amplification of bacterial (16S) and fungi (18S) DNA in sample	Extraction of DNA followed by PCR using universal 16S and 18S primers. Hours	Detects bacterial and fungal DNA.	List of microbes without context. Cannot distinguish live or dead. Relative abundance in each sample.	(Hilt and Ferrieri, 2022)
M-PCR	PCR, specific primers identify a panel of microbes	Amplified DNA, Hours to result.	Rapid, sensitive	List of microbes without context of infection, Only detects microbes specific to primer sets.	(Wojno et al., 2020; Hilt and Ferrieri, 2022)
M-PCR with Immune Markers of Infection	PCR, specific primers identify a panel of microbes coupled with immunoassays of markers of infection	MPCR panel coupled with immunoassays for immune markers.	Rapid, sensitive, links results to immune response, distinguishes urobiome from uropathogens	Only detects microbes specific to primer sets	(Akhlaghpour et al., 2024; Haley et al., 2024)
Shotgun Next Generation Metagenomic Sequencing	Extracts and sequences all DNA in clinical sample. Requires host DNA depletion to amplify signal.	DNA extraction from clinical sample, host depletion of DNA followed by shotgun metagenomic DNA sequencing	Detects all DNA: viruses, bacteriophages, eukaryotic microbes, bacteria, and human. Targeted metagenomics uses specific sequencing primers.	Expensive, time limited. Recent targeted approaches may revolutionize this approach	(Chang et al., 2025; Hilt and Ferrieri, 2022; Neugent et al., 2022)

**Table 3:** Incidence of known uropathogens using standard diagnostics and M-PCR.

Microbe	Gram stain	Oxygen tolerance	Uncomplicated UTI (% of cases)	Complicated UTI (% of cases)	M-PCR (% of cases)
*Escherichia coli*	Negative	Facultative Anaerobe	75 ^a^ 70 ^b^ 75 ^c^ 72.5 ^d^	65 ^a^ 65 ^b^ 65 ^c^ 55.4 ^d^	41^e^
*Klebsiella pneumoniae*	Negative	Aerobe	11 ^a^ 9.8 (*Klebsiella* spp)^b^ 6 (*Klebsiella* spp) ^c^ 5.1 ^d^	16 ^a^ 9.7 (*Klebsiella* spp)^b^ 8 (*Klebsiella* spp) ^c^ 8.9 ^d^	13 ^e^
*Enterococcus* spp	Positive	Facultative Anaerobe, Microaerophile	5.5 ^a^ 5.8 ^b^ 5.0 ^c^ 5.1 ^d^	10.5 ^a^ 4.7 ^b^ 11.0 ^c^ 8.9 ^d^	22 ^e^
*Staphylococcus saprophyticus*	Positive	Facultative Anaerobe, Microaerophile	5.5 ^a^ 5.5 (*Staphylococcus* spp) ^b^ 6.0 ^c^ 3.8 (CoNS) ^d^	NR ^a^ 7.3 (*Staphylococcus* spp) ^b^ NR ^c^ 2.5 (CoNS) ^d^	6 (CoNS) ^e^
*Proteus mirabilis*	Negative	Facultative Anaerobe	4.0 ^a^ 2.1 ^b^ 2.0 ^c^ 3.0 ^d^	4.0 ^a^ 2.2 ^b^ 2.0 ^c^ 4.4 ^d^	4 ^e^
*Pseudomonas aeruginosa*	Negative	Obligate Aerobe	2.5 ^a^ 1.0 ^b^ 1.0 ^c^ 2.9 ^d^	4.0 ^a^ 0.8 ^b^ 2.0 ^c^ 6.5 ^d^	4 ^e^
*Streptococcus* spp including GBS	Positive	Facultative Anaerobe	NR ^a^ 1.9 ^b^ 3.0 ^c^ 4.7 ^d^	NR ^a^ 3.9 ^b^ 2.0 ^c^ 4.2 ^d^	3 ^e^
*Candida* spp (Yeasts)	NA	Facultative Anaerobe	NR ^a^ 1.3 ^b^ 1.0 ^c^ 1.6 ^d^	NR ^a^ 2.9 ^b^ 3.0 ^c^ 2.8 ^d^	5 ^e^
*Staphylococcus aureus*	Positive	Facultative Anaerobe	NR ^a^ 5.5 (*Staphylococcus* spp) ^b^ 1.0 ^c^ 1.6 ^d^	NR ^a^ 7.3 (*Staphylococcus* spp) ^b^ 3.0 ^c^ 2.3 ^d^	1 ^e^
*Citrobacter* spp	Negative	Facultative Anaerobe	NR ^a^ 1.1 ^b^ NR ^c^ 1.6 ^d^	NR ^a^ 1.5 ^b^ NR ^c^ 2.8 ^d^	1.4 ^e^
*Enterobacter* spp	Negative	Facultative Anaerobe	NR ^a^ 1.0 ^b^ NR ^c^ 1.6 ^d^	NR ^a^ 1.4 ^b^ NR ^c^ 3.7 ^d^	3 ^e^
*Pseudomonas aeruginosa*	Negative	Obligate Aerobe	1.0 ^a^ 1.0 ^b^ 1.0 ^c^ 2.9 ^d^	2.9 ^a^ 0.8 ^b^ 2.0 ^c^ 6.5 ^d^	4 ^e^

a Adapted from Figure 2 (Mancuso et al., 2023).

b Adapted from **Figure 1** (Werneburg et al., 2023).

c Adapted from **Figure 1** (Flores-Mireles et al., 2015).

d Adapted from **Figure 1** (Gaston et al., 2021).

e Adapted from **Figure 1** (Haley et al., 2024).

NR, Not reported.

CoNS, Collectively known as coagulase negative staphylococcus (CoNS) and comprising *S. epidermidis*, *S. haemolyticus*, *S. lugdunensis*, *S. saprophyticus* (Moreland et al., 2023).

GBS, Group B *Streptococcus* (*Streptococcus agalactiae*) (Kline and Lewis, 2016).

NA, Not applicable.

Guidelines for common microbial contaminants in blood culture are available (Palavecino et al., 2024) and the Centers for Disease Control maintains a list of microbes that have been detected as commensals in UTI and blood infections (CDC, 2022). However, a list of urinary tract microbial contaminants is much more nebulous beyond listing niches (periurethral, epidermal, perianal, and vaginal), or assuming that microbes that are commensals in one niche are commensals in another (Blake and Doherty, 2006; LaRocco et al., 2015) (**Supplementary Tables 1**, **2**).

Thus, in current clinical diagnostic practice, if a sample contains mixed flora, it is the number of different microbes (≥ 2 or more at 10^4^ CFU/ml) that determines contamination and not the actual microbe unless it is a commonly recognized urinary pathogen (Bekeris et al., 2008; LaRocco et al., 2015; Sfeir and Hooton, 2018; Mancuso et al., 2023).

## 3 Limitations of diagnostic standard urine culture

Today, diagnosis of UTIs typically relies on patient symptoms and urinalysis. The latter uses urine dipstick testing and, in some cases, the standard urine culture (SUC) method (Chambliss and Van, 2022; Moreland et al., 2024; Werneburg et al., 2023) **Table 2**. Urine dipstick testing, frequently used to determine further testing, such as urine cultures is reviewed elsewhere (Moreland et al., 2024; Chambliss and Van, 2022). The limitations of SUC have been identified and are starting to impact current diagnostics (Price et al., 2017; Dixon et al., 2020; Wojno et al., 2020; Brubaker et al., 2023; Gleicher et al., 2024; Werneburg and Hsieh, 2024).

It is now well recognized that SUC under aerobic conditions (Gillespie et al., 1960), or even under 5% CO_2_, detects a limited number of microbes, almost all facultative anaerobes (Price et al., 2016, 2017; Wojno et al., 2020; Brubaker et al., 2023; Festa et al., 2023). As a result, reports based on SUC, including almost all literature to date, repeatedly document a constellation of the same microbes from the genera *Escherichia*, *Pseudomonas*, *Klebsiella*, *Proteus*, *Staphylococcus*, and *Enterococcus*, with *Escherichia coli* by far considered the predominant cause of UTI (**Table 3**) (Flores-Mireles et al., 2015; Kline and Lewis, 2016; Mancuso et al., 2023; Moreland et al., 2023; Werneburg et al., 2023; Timm et al., 2025). However, these results have been obtained because SUC was designed to detect fast growing, non-fastidious, facultative anaerobes, and thus fails to detect many other microbes. For example, a recent study directly compared SUC results to those of a multiplex polymerase chain reaction (M-PCR) panel for a cohort of 1,132 diagnosed UTI patients. M-PCR detected microbes in 823 of these patients, who also exhibited elevated infection-associated urine biomarkers (Haley et al., 2024). Of the 10 microbes most detected by M-PCR, only 4 were detected by SUC with 2 of those often not detected (Haley et al., 2024). Most striking was the failure of SUC to detect 3 of the 5 microbes most detected by M-PCR. These were the genera *Aerococcus* and *Actinotignum* and the Viridians group Streptococcus (including *S. anginosus*, *S. oralis*, and *S. gallolyticus* subsp. *pasteurianus* (formerly known as *Streptococcus pasteurianus*) (Doern and Burnham, 2010). Thus, except for *E. coli*, all known uropathogens (“the usual suspects”) represented only 13% or less of UTIs diagnosed with symptoms (Haley et al., 2024) (**Table 3**).

One outstanding issue with SUC has been the diagnosis of sterile pyuria, defined as positive for white blood cells but negative urine cultures in patients that report UTI symptoms (Wise and Schlegel, 2015; Horton et al., 2018; Cohen et al., 2019; Xu et al., 2024). Yet, a recent report suggests that *Actinotignum* (which SUC cannot detect but M-PCR diagnostics finds to be quite common) may be an underlying cause of sterile pyuria (Horton et al., 2018). Until recently, however, few had questioned the standard diagnostic method as flawed. As this standard method supports the established dogma that UTIs are caused by microbes arising from the gastrointestinal tract and, in females, vulvo-vagina reservoirs, it has, for the most part, gone unchallenged (Timm et al., 2025).

## 4 The urobiome “complication”

With the advent of DNA-based techniques (metagenomics) and enhanced culture methods (metaculturomics), the existence of female urethral and bladder microbiota has been confirmed with subtle differences existing between the two (Chen et al., 2020; Wang et al., 2023). Thus, we now know that the typical human urinary tract above the urinary sphincter is not sterile; instead, it contains an indigenous urinary microbiome (also known as the urobiome) (Price et al., 2020; Brubaker et al., 2021b; Du et al., 2024). We also know that the urobiome can have multiple healthy states (Pearce et al., 2014; Price et al., 2020; Jayalath and Magana-Arachchi, 2022; Joos et al., 2024). Moreover, many adult males and most adult females have a detectable urobiome without experiencing relevant urinary symptoms. Clearly, this does not mean we all have a UTI (Finucane, 2017). Consistent with clinical diagnosis of UTI, in the absence of relevant urinary symptoms, there is no “infection.” Diagnosis of a UTI requires that the patient exhibits at host response, and typically experiences symptoms (including urgency, frequency, urinary incontinence, and/or pain) (Anger et al., 2019).

Within any microbiome, microbes can be classified into 6 categories: non-pathogen (i.e., those that do not cause disease), pathogen (i.e., those that cause disease), commensal (i.e., those resident within the tissue and benefiting the host), symbiont (i.e., those resident within the tissue and benefiting both the host and the microbe), colonizer (i.e. resident within the tissue and may or may not cause disease), and pathobiont (i.e., resident within the tissue and generally beneficial but disease-causing under certain conditions) (Dey and Ray Chaudhuri, 2022). The urobiome has the full range of the 6 categories described above, including pathogens and pathobionts (Thomas-White et al., 2018; Du et al., 2024). Yet, most human beings do not have a clinical infection (i.e., UTI) even though pathogens or pathobionts are “citizens” of their urobiome community. An informative study enrolled heathy volunteers ≥ 65 years old without urinary symptoms as a comparison group for patients diagnosed with UTIs (Akhlaghpour et al., 2024). In that study, an M-PCR panel consistently detected the known uropathogens *E. coli* and *Enterococcus faecalis*, as well as the emerging uropathogens *Aerococcus urinae*, *Actinotignum schaalli*, and members of the Viridians group *Streptococcus* in healthy volunteers without urinary tract symptoms. In addition, few of these volunteers experienced an increase in infection-related immune markers, a hallmark of infection (Akhlaghpour et al., 2024). Furthermore, many adult females do not get UTIs. Finally, it is well known that UTIs can resolve spontaneously (Hoffmann et al., 2020; Barnes et al., 2021; Midby and Miesner, 2024). This implies that the indigenous urobiome together with both innate and adaptive immune responses can often restore urinary health and resolve infection.

Biomass influences microbial communities. The gastrointestinal tract is the best-known example of a high biomass microbial niche. In contrast, the urobiome has relatively low microbial biomass and thus, in some cases, urine samples yield culture-negative and DNA-based-negative test results (Hilt et al., 2014; Pearce et al., 2015; Neugent et al., 2020). An analogy would be a city block in the Bronx with 35,000 inhabitants versus a high plains plateau in Wyoming with sparse settlements. Within both types of communities, however, interactions occur among the residents. The same is true for ecological microcosms within the human microbiome.

If most contamination arises from periurethral, epidermal, perianal, and vaginal flora as suggested (LaRocco et al., 2015), it would be helpful to compare the bladder urobiome to these other niches. A comparison of gastrointestinal, vaginal and bladder microbiomes revealed that while all three niches were distinct from each other, there were similarities between the vagina and bladder microbiomes (Thomas-White et al., 2018; Du et al., 2024) (**Figure 1**). Furthermore, a recent survey of the skin microbiome lists the top ten taxa from four different niches (Byrd et al., 2018). Of the 22 different microbes identified among the ten most abundant taxa in four different regions of skin (dry, moist, sebaceous, and feet), 18 (82%) are also found in urine obtained directly from the bladder by transurethral catheterization (Thomas-White et al., 2018; Du et al., 2024) (**Supplementary Table 1**). Therefore, mere taxonomic identity (via Gram stain, oxygen tolerance, metabolic panel, and/or MALDI-TOF MS) cannot distinguish bladder residents from skin periurethral, perianal, and vaginal residents. To make such a distinction requires genome sequencing and complex bioinformatic analysis that determines whether two isolates are of the same lineage or not.

**Figure 1:**
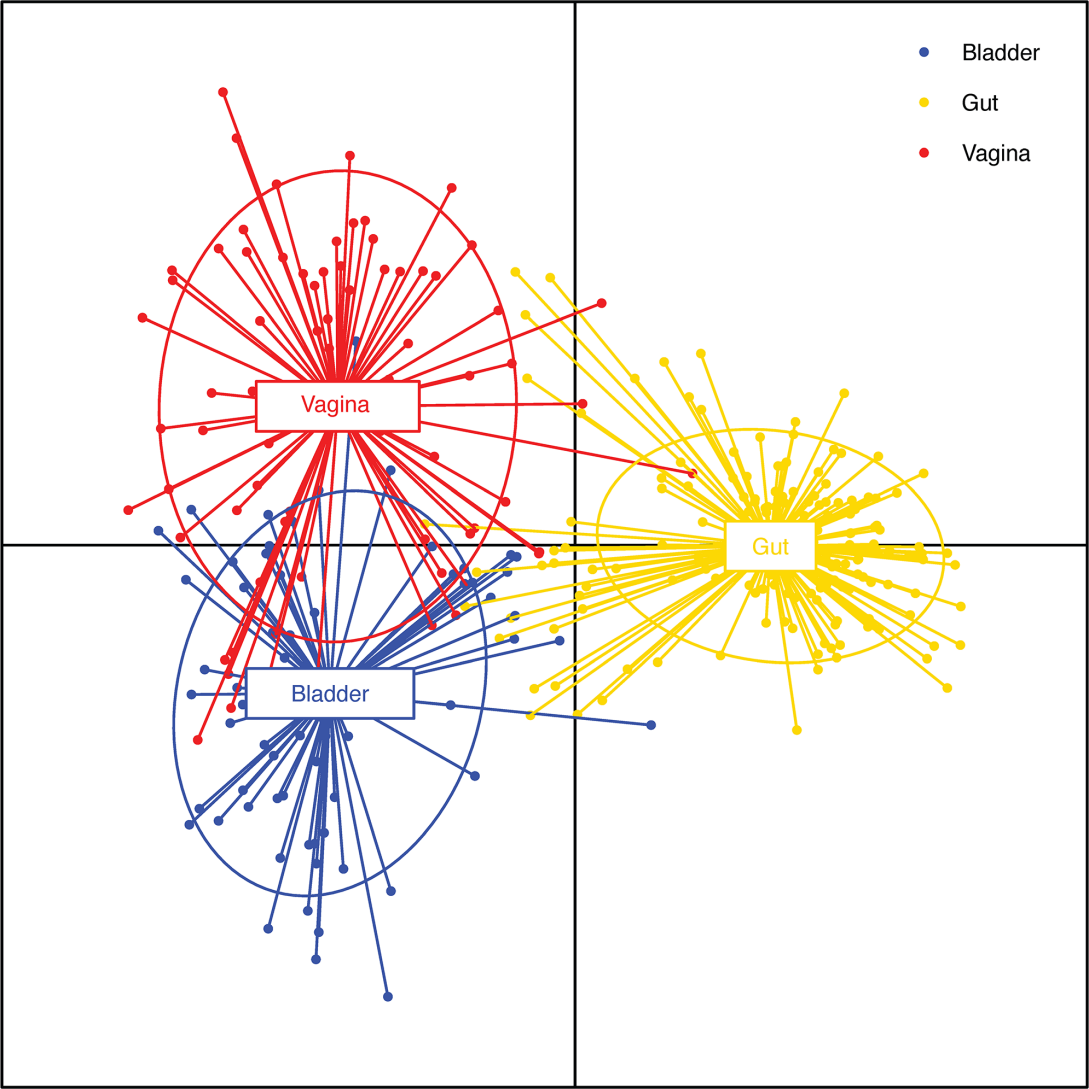
Comparison of bladder, vaginal and gut isolate functions. Discriminant analysis of principal components analysis of the functions of bacterial species isolated from asymptomatic individuals shared among 3 different niches: bladder (blue; n=68), vagina (red; n=74) and gut (yellow; n=175). The dots represent individual functions. The lines show the extent of those functions. Thus, a line that begins in the bladder centroid and ends in the vaginal centroid represents a function that is expressed by species that inhabit both the bladder and the vagina. Note the extensive overlap of functions in the bladder and vagina in contrast to the limited overlap between functions characteristic of the bladder or vagina and those of the gut. Adapted from (Du et al., 2024) (published under a Creative Commons license).

## 5 Polymicrobial infections

The history of polymicrobial infections dates to the late nineteenth century and W.D. Miller, a microbiologist and dentist who had worked in Robert Koch’s lab (Murray et al., 2014; Sedgley, 2004). By microscopy and characterization of pus from oral abscesses, he reported that the application of Koch’s postulates was not consistent with cultured microbes isolated from murine models. In fact, Miller obtained a more virulent response from the abscess pus than he did the cultured, recovered microbes. Armed with only culture conditions, a microscope and staining, Miller concluded that uncultivatable microbes worsened the infection (Murray et al., 2014). It was not until the availability of DNA sequence-based methods in the late twentieth and early twenty-first century that microbial ecology and polymicrobial infections were confirmed and appreciated in some human niches.

Thus, the concept of polymicrobial UTIs is not new but their existence has been confirmed with many examples reported in the late twentieth century (Siegman-Igra, 1994; Siegman-Igra et al., 1988, 1993; Siegman-IgraY, 1994). Yet, it may be useful to divide the studies on mixed cultures/polymicrobial infections into two groups: before and after the advent of DNA molecular techniques. The former was limited to urine sediment, urine culture, microscopy, Gram staining, and metabolic panels. Urine culture limited to SUC (most often aerobic) identified mostly facultative anaerobes (**Tables 3**, **4**). Consequently, this literature identified mostly Gram-negative rods as pathogens and excluded most Gram-positive rods and cocci as contaminants (with a few exceptions such as *Enterococcus* and coagulase negative *Staphylococcus* (CoNS), especially *S. saprophyticus*. Early studies on urosepsis and mixed (polymicrobial) cultures from patients revealed that matching cultures from urine and blood of the same microbe suggested that upper urinary tract infection had transitioned into the bloodstream. In one study, 716 bacteremic episodes were observed in 692 patients out of 52,012 admissions over 5 years (Siegman-Igra, 1994). Of these, 307 episodes in 303 patients were due to UTI. In this group, 198 had at least one microbe that was detected in both blood and urine culture (**Table 4**). These 198 urosepsis cases represented 194 patients (96 male, 98 female) with a mean age of 68 years. The most common microbe in monomicrobial infections was *Escherichia coli*; however, in polymicrobial infections, *Pseudomonas aeruginosa* was most predominant. *P. aeruginosa* also was among the microbes associated with fatal outcomes. In an earlier study of polymicrobial bacteremia, of 67 cases across multiple organ systems and causes, 46 percent were diagnosed with UTI (Siegman-Igra et al., 1988). Of the 67 cases, 28 died, with 42 percent diagnosed with UTI. While urosepsis is considered rare among the population, in neonates and patients 65 and older, it is a significant morbidity (Peach et al., 2016; Akhtar et al., 2021; Collaborators, GBD 2021 Antimicrobial Resistance, 2024).

**Table 4:** Characteristics of microbes from UTI with matching blood infections^a^ .

Microbe	Blood Culture^b^			Urine Culture^c^		
	Monomicrobial n (%)	Polymicrobial n (%)	Total n (%)	Monomicrobial n (%)	Polymicrobial n (%)	Total n (%)
*Escherichia coli*	77 (59)	32 (33)	109 (48)	77 (59)	43 (28)	120 (43)
*Klebsiella* spp^d^	21 (16)	19 (20)	40 (18)	21 (16)	29 (19)	50 (18)
*Proteus* spp^e^	15 (12)	18 (19)	33 (15)	15 (12)	30 (20)	45 (16)
*Pseudomonas* spp	8 (6)	16 (16)	24 (11)	8 (6)	25 (17)	33 (12)
*Enterococcus*	4 (3)	7 (7)	11 (5)	4 (3)	14 (9)	18 (6)
Other	5 (4)	5 (5)	10 (4)	5 (4)	11 (7)	16 (5)
Total	130 (100)	97 (100)	227 (100)	130 (100)	152 (100)	282 (100)

a Adapted from Table IV (Siegman-Igra, 1994) Used by permission.

b Blood culture: Bottles with tryptic soy broth, 5% CO_2_, 37C. Checked daily for growth, subcultured, stained after 24-48h.

c Urine culture: 5% sheep blood and MacConkey agar plates (loops with 0.01-0.001ml). Incubated 18-24h (aerobic).

d Including *Enterobacter* spp.

e Including *Morganella* spp and *Providencia* spp.

While traditional urinary pathogens are predominantly Gram-negative facultative anaerobes, Gram-positive microbes are also detected in patients diagnosed with UTI, particularly in polymicrobial infections (Kline and Lewis, 2016). While usually regarded as commensals, Gram-positive microbes’ pathogenic potential have been questioned (Clarke et al., 2010; Kline and Lewis, 2016; Leal et al., 2016). Yet, Gram-positive CoNS and *Enterococcus*, as well as emerging urinary pathogens such as *Aerococcus*, *Actinotignum*, *Gardnerella*, and *Corynebacteria* are found prevalently and abundantly in patients with UTI symptoms (Kline and Lewis, 2016; Moreland et al., 2023). Microaerophiles and anaerobes also have been observed (Legaria et al., 2022; Maskell, 1986) and cultures to rule out these microbes was suggested as part of diagnosis seventy years ago (Jawetz, 1953).

With the advent of metaculturomic methods (approaches designed to permit growth of typically uncultivated microbes) and metagenomic approaches (DNA-dependent methods that do not require growth), attempts (e.g., the human microbiome project) have sought to define the microbiota of various human niches, notably skin, respiratory tract, and the gastrointestinal tract, as well as the urogenital and reproductive tracts (Lloyd-Price et al., 2016; Joos et al., 2024). Lessons learned from the last two decades of research have taught us that there are multiple healthy states within niches that vary with sex, age and reproductive status (Lloyd-Price et al., 2016; Joos et al., 2024). Also, disease states are more complicated than originally anticipated by Koch and his postulated approach (Blevins and Bronze, 2010; Murray et al., 2014). A recent opinion paper questioned whether the urobiome has any impact on UTI, in part because Koch’s postulates have not been performed to determine whether any of the members of the newly identified urobiome cause disease symptoms (Werneburg and Southgate, 2024). Yet, Koch’s postulates (one organism, one infection, one cause of disease) cannot be applied to polymicrobial infections including UTIs (Ronald et al., 2008; Murray et al., 2014; Short et al., 2014). The request to use Koch’s postulates to validate the role of the urobiome (Werneburg and Southgate, 2024) is further complicated by the currently accepted tools that may no longer apply: a detection system (urine dipsticks, SUC) that misses many uropathogens (Moreland et al., 2024) and standards that define polymicrobial infections as contamination (Siegman-Igra et al., 1993; Siegman-IgraY, 1994; Bekeris et al., 2008).

One DNA-dependent method, multiplex PCR (M-PCR), allows quantitative, real-time detection of microbial DNA as long as a primer set is present to detect them (Moreland et al., 2024). Using M-PCR, a surprisingly high rate of polymicrobial specimens have been detected in older patients (≥ 65) with diagnosed UTI symptoms, ranging from 45 to 65 percent depending on simple or complex UTI, sex, and comorbidities (Vollstedt et al., 2020; Wojno et al., 2020; Korman et al., 2023; Wang et al., 2023; Akhlaghpour et al., 2024; Haley et al., 2024; Kardjadj et al., 2025). For example, in one study examining the differences of microbes based on catheterized (bladder) urine versus midstream void (entire urinary tract including urogenital areas), more polymicrobial infections were detected in midstream voided compared to catheter-collected samples (64.4% vs 45.7%, *p* < 0.0001) in females but the opposite in males (35.6% vs 47.0%, *p* = 0.002 (Wang et al., 2023). While M-PCR detected microbes that are not detected by SUC, the use of inflammatory markers of infection allowed the distinction of volunteers without relevant clinical symptoms from symptom-diagnosed UTI patients and stratifying those microbes into tiers based on abundance of occurrence (Akhlaghpour et al., 2024; Haley et al., 2024).

In the laboratory, specific microbes are studied in isolation using a reductionist approach. In the real world, communities of microbes make up an ecosystem that changes based on the predominating species and interactions between them (Murray et al., 2014; Short et al., 2014; Jayalath and Magana-Arachchi, 2022). These communities interact in synergy with residents by providing nutrients, soluble signaling factors, cell contact through adhesins, or in some cases facilitating antibiotic resistance (Ryan and Dow, 2008; Short et al., 2014; Murray et al., 2014; Gaston et al., 2021). Likewise, some microbes secrete molecules that eliminate their competition such as *Pseudomonas* (Gaston et al., 2021) or kill uropathogens such as commensal *Lactobacillus* (Abdul-Rahim et al., 2021; Johnson et al., 2022; Szczerbiec et al., 2022).

While polymicrobial results in the studies described above were reported as a group, the individual microbes within the subsets of polymicrobial infections were not identified. Instead, individual microbes were reported by taxon (Vollstedt et al., 2020; Wojno et al., 2020; Korman et al., 2023; Wang et al., 2023; Akhlaghpour et al., 2024; Haley et al., 2024). A reanalysis of the data from these studies could reveal associations of specific microbes that as a group or community are more pathogenic than as single isolates. In an effort to understand microbial ecology of polymicrobial isolates, one study examined 72 bacteria isolates collected from 23 patients diagnosed with polymicrobial UTI (de Vos et al., 2017). An ecological network analysis was developed, finding that most interactions clustered based on evolutionary relatedness. Eight complete communities of four different microbes were found, four of which were predicted to be stable and four not stable (de Vos et al., 2017). The isolates used for this study were originally collected using standard culture techniques (Croxall et al., 2011), which may explain the predominance of classical uropathogens. Expanding and extending this concept, another study examined pathogens and bladder commensal isolates grown under urine-like conditions and the effects different microbes had on the community (Zandbergen et al., 2021). Artificial urine media conditioned by commensals had effects on growth of uropathogens and vice versa. This early attempt at gaining insights into the complexities of urobiome ecology shows that while a microbe in isolation may have certain growth characteristics, the community - through direct physical interaction, direct interaction with the host, or secretion/excretion of signaling molecules or metabolites, - may exhibit a different response than a single microbe in monoculture (Heidrich et al., 2022; Short et al., 2014; Murray et al., 2014).

## 6 Future directions: a call to action

To advance diagnosis, we suggest the following:

1. Reexamine standards for urine collection/culture contamination to ensure accurate sampling, taking into consideration the new knowledge concerning the urobiome. A recent scoping review highlights the issues, especially the lack of consensus among guidelines concerning urine culture thresholds for UTI and the reliance upon dated and sparse evidence for current standards (Hilt et al., 2023). To achieve accuracy, we should recognize that urine samples must be processed immediately or be stored under conditions that do not permit microbial growth (LaRocco et al., 2015).
2. Recognize the shortcomings of SUC. There is now sufficient data in the literature to adjudicate the accuracy of this method. It should be examined, weighing its strengths and acknowledging its weaknesses (Price et al., 2017; Wojno et al., 2020; Xu et al., 2021; Festa et al., 2023; Brubaker et al., 2023; Gleicher et al., 2024). Besides its ability to detect only a limited group of facultative anaerobes, time is also a consideration as re-sampling and re-culturing requires time that may allow an infection to progress.
3. Recognize the presence and importance of polymicrobial UTI. The literature provides evidence that these infections are more prevalent than previously believed and misdiagnosis as contamination should be considered and re-evaluated. Ultimately, clinical diagnostic pathways might be modified to diagnose these infections before they can progress to upper tract UTI and potentially urosepsis (Siegman-Igra, 1994; Peach et al., 2016; Akhtar et al., 2021).
4. Recognize the importance of redefining contamination, acknowledging the existence of polymicrobial infection. Critical for antibiotic stewardship is the development of accurate and rapid diagnostics that not only detect microbes but also determine if they are involved in a host immune response. The increase in antibiotic resistance and future predictions are sobering (Collaborators, GBD 2021 Antimicrobial Resistance, 2024). Proper and appropriate use of antibiotics to treat UTI not only affects the health of patients but preserves critical antibiotics for those who need them (Simoni et al., 2024).
5. Take a lesson from other organ systems and diseases (Murray et al., 2014; Short et al., 2014), and ask how diagnostic tools can be improved and new tools developed or implemented to characterize microbial communities in polymicrobial infections/mixed cultures. One way to accomplish this would be to reanalyze data from polymicrobial samples to identify interaction networks and determine if any groups of microbes are more commonly associated with symptoms or the lack thereof (Vollstedt et al., 2020; Wang et al., 2023; Akhlaghpour et al., 2024; Haley et al., 2024).
6. Finally, the concept of contamination must be reevaluated in the context of the new knowledge that the urobiome exists. Urine is not sterile above the urethral sphincter and the existence of communities containing both commensal, non-pathogenic microbes and potential uropathogens should be acknowledged. While dysbiosis is generally a concept foreign to the UTI literature, it needs to be recognized, and diagnostics updated to reflect the current science (Price et al., 2017; Jayalath and Magana-Arachchi, 2022; Simoni et al., 2024). Koch’s postulates are invalid in polymicrobial systems (Murray et al., 2014; Short et al., 2014) but unfortunately continue to persist as a consequence of the dogma that single microbes are causative of disease (Blevins and Bronze, 2010; Werneburg and Southgate, 2024).

The danger of dismissing species considered to be routine contaminants, such as members of the genus *Corynebacterium*, can lead to dismissing pathogens like *C. urealyticium*, which is both nitrate negative by urine dipstick and grows slowly under SUC conditions. Yet, while PCR testing is much more rapid than culture (Dixon et al., 2020; Gleicher et al., 2024; Zering and Stohs, 2024), without some reference to host immune response (indicative of infection), the practicing clinician is left with 10–20 names on a page with little clue what to do next (Zering and Stohs, 2024; Xu et al., 2021). Fortunately, one test correlates an M-PCR panel of 30 microbes with biomarkers of inflammation (Akhlaghpour et al., 2024; Haley et al., 2024). While this is but one study with older patients, it opens the opportunity for researchers to examine other groups (e.g., different age groups, males versus females, or pregnant versus non-pregnant individuals.It is likely the differences encountered will require tailored treatments depending on the type of patient.

So, what should we do with this additional information? We foresee both scientific and clinical paths. As scientists broaden the view from “identify the pathogen and kill it” to “understand the ecology,” new insights into therapies should arise that parallel those of the oncological research and clinical community that has spent decades working to better understand, diagnose, and treat cancers. Their recognition that the old diagnostics and treatment algorithms were insufficient has led to precision diagnostics, targeted treatments, and better outcomes for patients. We should do the same for our patients. Teams of scientists and clinicians have begun this process; others should join the effort.

Where does this leave clinical microbiologists and clinicians who deal daily with the quandary of accurate diagnosis balanced by microbial stewardship? Even though the human microbiome project began two decades ago, the translation of that research into clinical practice is just beginning (Gilbert et al., 2025). In other niches, where the research is more advanced, there have been three stages of translations into the clinic. The first stage involves identification of microbiota and normal (healthy) microbial ecology. This process leads to understanding the mechanisms underlying dysbiosis and symptoms/disease. The second stage develops metrics associating taxa, host response, and disease characteristics that lead to biomarkers and new diagnostics. Finally, armed with this information, strategies are developed to modify the microbiota to restore a normal, healthy state (Gilbert et al., 2025).

Our community is still in the earlier stages of this process, having shown that the urobiome exists and obtained evidence that it is associated with health and disease. Progress on the second stage has begun but there is much to do. To be consistent with developing science, clinical microbiologists must challenge current standards concerning urine culture contamination (Bekeris et al., 2008). Also, the incidence of polymicrobial infection must be established across diverse populations (e.g. age, sex, nosocomial versus outpatient status). For their part, clinicians should be aware of the older diagnostics flaws and help progress new diagnostic developments with the goal of a rapid, accurate test that identifies both microbes and host response. For diagnosis of UTI, the host response could be monitored by inflammatory biomarkers (Akhlaghpour et al., 2024). In the meantime, clinicians can be open to emerging new knowledge and new diagnostics. Their intellectual preparation to use that knowledge to make their best clinical decision will be critical for improved patient care. Finally, all should recognize that better diagnostics and better understanding of their results are key to antibiotic stewardship as the convenience of empirical treatment, or the use of flawed culture methods ultimately will deprive those who need antibiotics to survive in an increasingly antibiotic-resistant clinical environment (Collaborators, GBD 2021 Antimicrobial Resistance, 2024; Simoni et al., 2024).

## 7 Conclusions

In this review, we have presented the current diagnostic state of urine contamination and discussed the limitations of current diagnostic techniques, such as SUC. We have reviewed the evidence for polymicrobial UTI, raising doubt concerning the appropriateness of applying Koch’s postulates and recognizing the consequences of potential missed diagnosis. The challenges for clinical microbiologists, clinicians and research scientists are to question the current dogmas, address polymicrobial infections, and work to define the microbial ecology of the urinary tract. The future is bright as multidisciplinary collaboration offers cross-pollinating efforts to improve patient care and quality of life.

## Supplementary material

Supplementary Table 1 Most abundant skin microbes by region/niche^a^ and incidence in urobiome^b^. ^a^ From the top ten most abundant microbes in four different skin regions (Byrd et al., 2018). Note that some microbes are abundant in multiple regions (for ex., *Corynebacterium tuberculostearicum* and *Staphylococcus epidermidis*). Across four regions with ten microbes each, there are 22 unique taxa. ^b^ Microbes in bold type were found in the human female urobiome (Du et al., 2024) as determined by sequencing of genomes from isolates from catheterized urinary bladder urine. ^c^ Skin regions/niches are defined as follows: Dry: Hypothenar palm and volar forearm. Moist: Nare, antecubital fossa, inguinal crease, interdigital web, and popliteal fossa. Sebaceous: Alar crease, cheek, glabella, external auditory canal, manubrium, retroauricular crease, occiput, back. Feet:|Toe web space, toenail, andplantar heel. ^d^ Bacterial Diversity Database. See (Schober et al., 2025). ^e^ Also known as *Cutibacterium acnes.*

Supplementary Table 2 Most abundant vaginal microbes^a^ and incidence in urobiome^b^. ^a^ Ravel et al., 2011 evaluated vaginal microflora and vaginal pH in 396 asymptomatic sexually active women representing four ethnic groups (white, black, Hispanic, and Asian) by pyrosequencing of 16S rRNA genes. *Lactobacillus* species dominated four groups, but one group accounting for 27% of women (termed as group IV community state type) lacked significant lactobacilli and was heterogeneous with higher proportion of strictly anaerobic bacteria including genera *Gardnerella*, *Prevotella*, *Atopobium*, as well as *Dialister*, *Megasphaera*, *Peptoniphilus*, *Sneathia*, *Eggerthella*, *Aerococcus*, *Finegoldia*, and *Mobiluncus*. This latter group was over-represented in black and Hispanic women (Ravel et al., 2011). ^b^ Microbes in bold type were found in the human female urobiome (Du et al., 2024) as determined by sequencing of genomes from isolates from catheterized urinary bladder urine. ^c^ For bacterial vaginosis, see (Saraf et al., 2021) ^d^ Bacterial Diversity Database. See (Schober et al., 2025). ^e^ *Atopobium vaginae* is now known as *Fannyhessea vaginae* (Nouioui et al., 2018).

## References

[B1] Abdul-Rahim O. Wu Q. Pistone G Price T. K. Diebel K. Wolfe AJ Bugni T. S. (2021). Phenyl-lactic acid is an active ingredient in bactericidal supernatants of lactobacillus crispatus. J. Bacteriol. 203, e0036021. doi: 10.1128/JB.00360-21 34280003 PMC8425402

[B2] Akhlaghpour M. Haley E Parnell L. Luke N. Mathur M. Festa R. A. Percaccio M.. (2024). Urine biomarkers individually and as a consensus model show high sensitivity and specificity for detecting UTIs. BMC Infect. Dis. 24, 153. doi: 10.1186/s12879-024-09044-2 38297221 PMC10829179

[B3] Akhtar A. Ahmad Hassali M. A. Zainal H. Ali I. Khan A. H. (2021). A cross-sectional assessment of urinary tract infections among geriatric patients: prevalence, medication regimen complexity, and factors associated with treatment outcomes. Front. Public Health 9, 657199. doi: 10.3389/fpubh.2021.657199 34733812 PMC8558341

[B4] Anger J. Lee U. Ackerman A. L. Chou R. Chughtai B. Clemens J. Q.. (2019). Recurrent uncomplicated urinary tract infections in women: AUA/CUA/SUFU guideline. J. Urol. 202, 282–289. doi: 10.1097/JU.0000000000000296 31042112

[B5] Armbruster C. E. Smith S. N. Johnson A. O. DeOrnellas V. Eaton K. A. Yep A.. (2017). The Pathogenic Potential of Proteus mirabilis Is Enhanced by Other Uropathogens during Polymicrobial Urinary Tract Infection. Infect. Immun. 85, e00808–e00816. doi: 10.1128/IAI.00808-16 27895127 PMC5278182

[B6] Asscher A. W. Sussman M. Waters W. E. Davis R. H. Chick S. (1966). Urine as a medium for bacterial growth. Lancet 2, 1037–1041. doi: 10.1016/S0140-6736(66)92023-X 4162501

[B7] Barnes H. C. Wolff B. Abdul-Rahim O. Harrington A. Hilt E. E. Price T. K.. (2021). A randomized clinical trial of standard versus expanded cultures to diagnose urinary tract infections in women. J. Urol. 206, 1212–1221. doi: 10.1097/JU.0000000000001949 34184930

[B8] Bekeris L. G. Jones B. A. Walsh M. K. Wagar E. A. (2008). Urine culture contamination: a College of American Pathologists Q-Probes study of 127 laboratories. Arch. Pathol. Lab. Med. 132, 913–917. doi: 10.5858/2008-132-913-UCCACO 18517272

[B9] Blake D. R. Doherty L. F. (2006). Effect of perineal cleansing on contamination rate of mid-stream urine culture. J. Pediatr. Adolesc. Gynecol. 19, 31–34. doi: 10.1016/j.jpag.2005.11.003 16472726

[B10] Blevins S. M. Bronze M. S. (2010). 'Robert Koch and the 'golden age' of bacteriology. Int. J. Infect. Dis. 14, e744–e751. doi: 10.1016/j.ijid.2009.12.003 20413340

[B11] Brubaker L. Chai T. C. Horsley H. Moreland R. B. Khasriya R. Wolfe A. J. (2023). 'Tarnished gold—the “standard” urine culture: reassessing the characteristics of a criterion standard for detecting urinary microbes. Front. Urol. 3. doi: 10.3389/fruro.2023.1206046 PMC12327316 40778073

[B12] Brubaker L. Gourdine J. F. Siddiqui N. Y. Holland A. Halverson T. Limeria R.. (2021a). Forming consensus to advance urobiome research. mSystems 6, e0137120. doi: 10.1128/msystems.01371-20 34282932 PMC8409733

[B13] Brubaker L. Putonti C. Dong Q. Wolfe A. J. (2021b). The human urobiome. Mamm. Genome 32, 232–238. doi: 10.1007/s00335-021-09862-8 33651197

[B14] Byrd A. L. Belkaid Y. Segre J. A. (2018). The human skin microbiome. Nat. Rev. Microbiol. 16, 143–155. doi: 10.1038/nrmicro.2017.157 29332945

[B15] CDC (2022) National healthcare safety network master organism list. (Online database). Available at: https://www.cdc.gov/nhsn/xls/master-organism-com-commensals-lists.xlsx.

[B16] Chambliss A. B. Van T. T. (2022). Revisiting approaches to and considerations for urinalysis and urine culture reflexive testing. Crit. Rev. Clin. Lab. Sci. 59, 112–124. doi: 10.1080/10408363.2021.1988048 34663175

[B17] Chang Z. Deng J. Zhang J. Wu H. Wu Y. Bin L.. (2025). Rapid and accurate diagnosis of urinary tract infections using targeted next-generation sequencing: A multicenter comparative study with metagenomic sequencing and traditional culture methods. J. Infect. 90, 106459. doi: 10.1016/j.jinf.2025.106459 40058503

[B18] Chen Y. B. Hochstedler B. Pham T. T. Acevedo-Alvarez M. Mueller E. R. Wolfe A. J. (2020). The urethral microbiota: A missing link in the female urinary microbiota. J. Urol. 204, 303–309. doi: 10.1097/JU.0000000000000910 32118507

[B19] Clarke T. M. Citron D. M. Towfigh S. (2010). The conundrum of the gram-positive rod: are we missing important pathogens in complicated skin and soft-tissue infections? A case report and review of the literature. Surg. Infect. (Larchmt). 11, 65–72. doi: 10.1089/sur.2008.085 19803730

[B20] Cohen J. E. Yura E. M. Chen L. Schaeffer A. J. (2019). Predictive utility of prior negative urine cultures in women with suspected recurrent uncomplicated urinary tract infections. J. Urol. 202, 979–985. doi: 10.1097/JU.0000000000000325 31063050

[B21] Collaborators, GBD 2021 Antimicrobial Resistance (2024). Global burden of bacterial antimicrobial resistance 1990–2021: a systematic analysis with forecasts to 2050. Lancet 404 (10459), 1199–1226. doi: 10.1016/S0140-6736(24)01867-1 39299261 PMC11718157

[B22] Croxall G. Weston V. Joseph S. Manning G. Cheetham P. McNally A. (2011). Increased human pathogenic potential of Escherichia coli from polymicrobial urinary tract infections in comparison to isolates from monomicrobial culture samples. J. Med. Microbiol. 60, 102–109. doi: 10.1099/jmm.0.020602-0 20947667

[B23] Deen N. S. Ahmed A. Tasnim N. T. Khan N. (2023). Clinical relevance of expanded quantitative urine culture in health and disease. Front. Cell Infect. Microbiol. 13, 1210161. doi: 10.3389/fcimb.2023.1210161 37593764 PMC10428011

[B24] de Vos M. G. J. Zagorski M. McNally A. Bollenbach T. (2017). Interaction networks, ecological stability, and collective antibiotic tolerance in polymicrobial infections. Proc. Natl. Acad. Sci. U. S. A 114, 10666–10671. doi: 10.1073/pnas.1713372114 28923953 PMC5635929

[B25] Dey P. Ray Chaudhuri S. (2022). The opportunistic nature of gut commensal microbiota. Crit. Rev. Microbiol. 49, 739–763. doi: 10.1080/1040841X.2022.2133987 36256871

[B26] Dixon M. Sha S. Stefil M. McDonald M. (2020). Is it time to say goodbye to culture and sensitivity? The case for culture-independent urology. Urology 136, 112–118. doi: 10.1016/j.urology.2019.11.030 31786305

[B27] Doern C. D. Burnham C. A. (2010). It's not easy being green: the viridans group streptococci, with a focus on pediatric clinical manifestations. J. Clin. Microbiol. 48, 3829–3835. doi: 10.1128/JCM.01563-10 20810781 PMC3020876

[B28] Du J. Khemmani M. Halverson T. Ene A. Limeira R. Tinawi L.. (2024). Cataloging the phylogenetic diversity of human bladder bacterial isolates. Genome Biol. 25, 75. doi: 10.1186/s13059-024-03216-8 38515176 PMC10958879

[B29] Festa R. A. Luke N. Mathur M. Parnell L. Wang D. Zhao X.. (2023). A test combining multiplex-PCR with pooled antibiotic susceptibility testing has high correlation with expanded urine culture for detection of live bacteria in urine samples of suspected UTI patients. Diagn. Microbiol. Infect. Dis. 107, 116015. doi: 10.1016/j.diagmicrobio.2023.116015 37499607

[B30] Finucane T. E. (2017). Urinary tract infection"-requiem for a heavyweight. J. Am. Geriatr. Soc. 65, 1650–1655. doi: 10.1111/jgs.2017.65.issue-8 28542707

[B31] Flores-Mireles A. L. Walker J. N. Caparon M. Hultgren S. J. (2015). Urinary tract infections: epidemiology, mechanisms of infection and treatment options. Nat. Rev. Microbiol. 13, 269–284. doi: 10.1038/nrmicro3432 25853778 PMC4457377

[B32] Friedmann H. C. (2014). Escherich and escherichia. EcoSal. Plus. 6, 133–196. doi: 10.1128/ecosalplus.esp-0025-2013 26442939

[B33] Gaston J. R. Johnson A. O. Bair K. L. White A. N. Armbruster C. E. (2021). Polymicrobial interactions in the urinary tract: is the enemy of my enemy my friend? Infect. Immun. 89. doi: 10.1128/IAI.00652-20 33431702

[B34] Gilbert J. A. Azad M. B. Bäckhed F. Blaser M. J. Byndloss M. Chiu C. Y.. (2025). Clinical translation of microbiome research. Nat. Med. 31, 1099–1113. doi: 10.1038/s41591-025-03615-9 40217076

[B35] Gillespie W. A. Linton K. B. Miller A. Slade N. (1960). The diagnosis, epidemiology and control of urinary infection in urology and gynaecology. J. Clin. Pathol. 13, 187–194. doi: 10.1136/jcp.13.3.187 13850096 PMC480047

[B36] Gleicher S. Karram M. Wein A. J. Dmochowski R. R. (2024). Recurrent and complicated urinary tract infections in women: Utility of advanced testing to enhance care. Neurourol. Urodyn. 43, 161–166. doi: 10.1002/nau.25280 37822027

[B37] Haley E. Luke N. Mathur M. Festa R. A. Wang J. Jiang Y.. (2024). The prevalence and association of different uropathogens detected by M-PCR with infection-associated urine biomarkers in urinary tract infections. Res. Rep. Urol. 16, 19–29. doi: 10.2147/RRU.S443361 38221993 PMC10787514

[B38] Hansen M. A. Valentine-King M. Zoorob R. Schlueter M. Matas J. L. Willis S. E.. (2022). Prevalence and predictors of urine culture contamination in primary care: A cross-sectional study. Int. J. Nurs. Stud. 134, 104325. doi: 10.1016/j.ijnurstu.2022.104325 35914376 PMC10513105

[B39] Heidrich V. Inoue L. T. Asprino P. F. Bettoni F. Mariotti A. C. H. Bastos D. A.. (2022). Choice of 16S ribosomal RNA primers impacts male urinary microbiota profiling. Front. Cell Infect. Microbiol. 12, 862338. doi: 10.3389/fcimb.2022.862338 35531325 PMC9069555

[B40] Hilt E. E. Ferrieri P. (2022). Next generation and other sequencing technologies in diagnostic microbiology and infectious diseases. Genes (Basel). 13, 1566–1590. doi: 10.3390/genes13091566 36140733 PMC9498426

[B41] Hilt E. E. McKinley K. Pearce M. M. Rosenfeld A. B. Zilliox M. J. Mueller E. R.. (2014). Urine is not sterile: use of enhanced urine culture techniques to detect resident bacterial flora in the adult female bladder. J. Clin. Microbiol. 52, 871–876. doi: 10.1128/JCM.02876-13 24371246 PMC3957746

[B42] Hilt E. E. Parnell L. K. Wang D. Stapleton A. E. Lukacz E. S. (2023). Microbial threshold guidelines for UTI diagnosis: A scoping systematic review. Pathol. Lab. Med. Int. 15, 43–63. doi: 10.2147/PLMI.S409488

[B43] Hoffmann T. Peiris R. Mar C. D. Cleo G. Glasziou P. (2020). Natural history of uncomplicated urinary tract infection without antibiotics: a systematic review. Br. J. Gen. Pract. 70, e714–ee22. doi: 10.3399/bjgp20X712781 32958533 PMC7510849

[B44] Hort E. C. (1914). The sterility of normal urine in man. J. Hyg. (Lond). 14, 509–516. doi: 10.1017/S0022172400006021 20474594 PMC2206779

[B45] Horton L. E. Mehta S. R. Aganovic L. Fierer J. (2018). Actinotignum schaalii infection: A clandestine cause of sterile pyuria? Open Forum Infect. Dis. 5, ofy015. doi: 10.1093/ofid/ofy015 29450211 PMC5808804

[B46] Jawetz E. (1953). Urinary tract infections; problems in medical management. California. Med. 79, 99–102. 13067022 PMC1522012

[B47] Jayalath S. Magana-Arachchi D. (2022). 'Dysbiosis of the human urinary microbiome and its association to diseases affecting the urinary system. Indian J. Microbiol. 62, 133–166. doi: 10.1007/s12088-021-00991-x PMC8980184 35462710

[B48] Johnson J. A. Delaney L. F. Ojha V. Rudraraju M. Hintze K. R. Siddiqui N. Y.. (2022). Commensal urinary lactobacilli inhibit major uropathogens *in vitro* with heterogeneity at species and strain level. Front. Cell Infect. Microbio. 12, 870603. doi: 10.3389/fcimb.2022.870603 PMC9260849 35811675

[B49] Joos R. Boucher K. Lavelle A. Arumugam M. Blaser M. J. Claesson M. J.. (2024). Examining the healthy human microbiome concept. Nat. Rev. Microbiol. 23 (3), 192–205. doi: 10.1038/s41579-024-01107-0 39443812

[B50] Kardjadj M. Chang T. W. Chavez R. Derrick D. Spangler F. L. Priestly I. P.. (2025). The clinical validity and utility of PCR compared to conventional culture and sensitivity testing for the management of complicated urinary tract infections in adults: A secondary (*Ad hoc*) analysis of pathogen detection, resistance profiles, and impact on clinical outcome. Microorganisms 13, 949. doi: 10.3390/microorganisms13040949 40284785 PMC12029264

[B51] Kavuru V. Vu T. Karageorge L. Choudhury D. Senger R. Robertson J. (2020). Dipstick analysis of urine chemistry: benefits and limitations of dry chemistry-based assays. Postgrad. Med. 132, 225–233. doi: 10.1080/00325481.2019.1679540 31609156

[B52] Khasriya R. Sathiananthamoorthy S. Ismail S. Kelsey M. Wilson M. Rohn J. L.. (2013). Spectrum of bacterial colonization associated with urothelial cells from patients with chronic lower urinary tract symptoms. J. Clin. Microbiol. 51, 2054–2062. doi: 10.1128/JCM.03314-12 23596238 PMC3697662

[B53] Kline K. A. Lewis A. L. (2016). Gram-positive uropathogens, polymicrobial urinary tract infection, and the emerging microbiota of the urinary tract. Microbiol. Spect. 4, 10.1128. doi: 10.1128/microbiolspec.UTI-0012-2012 PMC4888879 27227294

[B54] Korman H. J. Baunoch D. Luke N. Wang D. Zhao X. Levin M.. (2023). 'A diagnostic test combining molecular testing with phenotypic pooled antibiotic susceptibility improved the clinical outcomes of patients with non-E. coli or polymicrobial complicated urinary tract infections. Res. Rep. Urol. 15, 141–147. doi: 10.2147/RRU.S404260 37151752 PMC10162393

[B55] LaRocco M. T. Franek J. Leibach E. K. Weissfeld A. S. Kraft C. S. Sautter R. L.. (2015). Effectiveness of preanalytic practices on contamination and diagnostic accuracy of urine cultures: a laboratory medicine best practices systematic review and meta-analysis. Clin. Microbiol. Rev. 29, 105–147. doi: 10.1128/CMR.00030-15 PMC4771218 26598386

[B56] Leal S. M. Jr Jones M. Gilligan P. H. (2016). Clinical significance of commensal gram-positive rods routinely isolated from patient samples. J. Clin. Microbiol. 54, 2928–2936. doi: 10.1128/JCM.01393-16 27629905 PMC5121381

[B57] Legaria M. C. Barberis C. Famiglietti A. De Gregorio S. Stecher D. Rodriguez C. H.. (2022). Urinary tract infections caused by anaerobic bacteria. Utility of anaerobic urine culture. Anaerobe 78, 102636. doi: 10.1016/j.anaerobe.2022.102636 36210609

[B58] Lloyd-Price J. Abu-Ali G. Huttenhower C. (2016). The healthy human microbiome. Genome Med. 8, 51. doi: 10.1186/s13073-016-0307-y 27122046 PMC4848870

[B59] Mancuso G. Midiri A. Gerace E. Marra M. Zummo S. Biondo C. (2023). Urinary tract infections: the current scenario and future prospects. Pathogens 12, 623–640. doi: 10.3390/pathogens12040623 37111509 PMC10145414

[B60] Marple C. D. (1941). The frequency and character of urinary tract infections in a group of unselected women. Ann. Internal Med. 14, 2220–2239. doi: 10.7326/0003-4819-14-12-2220

[B61] Maskell R. (1986). Are fastidious organisms an important cause for dysuria and frequency- the case for (London, UK: John Wiley and Sons).

[B62] Maskell R. A. (1988). Pathogenesis, consequences and natural history of urinary tract infection (London, UK: Edward Arnold).

[B63] McFadyen I. R. Eykyn S. J. (1968). Suprapubic aspiration of urine in pregnancy. Lancet 1, 1112–1114. doi: 10.1016/S0140-6736(68)90185-2 4171842

[B64] Midby J. S. Miesner A. R. (2024). Delayed and non-antibiotic therapy for urinary tract infections: A literature review. J. Pharm. Pract. 37, 212–224. doi: 10.1177/08971900221128851 36134708

[B65] Moreland R. B. Brubaker L. Tinawi L. Wolfe A. J. (2024). Rapid and accurate testing for urinary tract infection: new clothes for the emperor. Clin. Microbiol. Rev. 38, e0012924. doi: 10.1128/cmr.00129-24 39641639 PMC11905368

[B66] Moreland R. B. Choi B. I. Geaman W. Hochstedler-Kramer B. R. Gonzalez C. John J.. (2023). Beyond the usual suspects: emerging uropathogens in the microbiome age. Front. Urol. 3. doi: 10.3389/fruro.2023.1212590 PMC12327349 40778068

[B67] Mundt L. A. Shanahan K. (2010). Chapter 4. Chemical Analysis of Urine (Philadelphia, PA: Wolters-Kluwer/Lippincott Williams & Wiliams).

[B68] Murray J. L. Connell J. L. Stacy A. Turner K. H. Whiteley M. (2014). Mechanisms of synergy in polymicrobial infections. J. Microbiol. 52, 188–199. doi: 10.1007/s12275-014-4067-3 24585050 PMC7090983

[B69] Neugent M. L. Hulyalkar N. V. Nguyen V. H. Zimmern P. E. De Nisco N. J. (2020). Advances in understanding the human urinary microbiome and its potential role in urinary tract infection. mBio 11, 100753–100770. doi: 10.1128/mBio.00218-20 PMC7188990 32345639

[B70] Neugent M. L. Kumar A. Hulyalkar N. V. Lutz K. C. Nguyen V. H. Fuentes J. L.. (2022). Recurrent urinary tract infection and estrogen shape the taxonomic ecology and function of the postmenopausal urogenital microbiome. Cell Rep. Med. 3, 100753. doi: 10.1016/j.xcrm.2022.100753 36182683 PMC9588997

[B71] Nouioui I. Carro L. García-López M. Meier-Kolthoff J. P. Woyke T. Kyrpides N. C.. (2018). Genome-based taxonomic classification of the phylum actinobacteria. Front. Microbiol. 9, 2007. doi: 10.3389/fmicb.2018.02007 30186281 PMC6113628

[B72] Nunn K. L. Forney L. J. (2016). Unraveling the dynamics of the human vaginal microbiome'. Yale J. Biol. Med. 89, 331–337. 27698617 PMC5045142

[B73] O'Leary B. D. Armstrong F. M. Byrne S. Talento A. F. O'Coigligh S. (2020). The prevalence of positive urine dipstick testing and urine culture in the asymptomatic pregnant woman: A cross-sectional study. Eur. J. Obstet. Gynecol. Reprod. Biol. 253, 103–107. doi: 10.1016/j.ejogrb.2020.08.004 32862029

[B74] Pagan L. Ederveen R. A. M. Huisman B. W. Schoones J. W. Zwittink R. D. Schuren F. H. J.. (2021). The human vulvar microbiome: A systematic review. Microorganisms. 9 (12), 2568. doi: 10.3390/microorganisms91225 34946169 PMC8705571

[B75] Palavecino E. L. Campodónico V. L. She R. C. (2024). Laboratory approaches to determining blood culture contamination rates: an ASM laboratory practices subcommittee report. J. Clin. Microbiol. 262 (2), e0102823. doi: 10.1128/jcm.01028-23 PMC10865823 38051070

[B76] Park M. G. Cho S. Oh M. M. (2023). Menopausal changes in the microbiome-A review focused on the genitourinary microbiome. Diagn. (Basel). 13, 1193. doi: 10.3390/diagnostics13061193 PMC10047399 36980501

[B77] Peach B. C. Garvan G. J. Garvan C. S. Cimiotti J. P. (2016). Risk factors for urosepsis in older adults: A systematic review. Gerontol. Geriatr. Med. 2, 2333721416638980. doi: 10.1177/2333721416638980 28138493 PMC5119864

[B78] Pearce M. M. Hilt E. E. Rosenfeld A. B. Zilliox M. J. Thomas-White K. Fok C.. (2014). The female urinary microbiome: a comparison of women with and without urgency urinary incontinence. mBio 5, e01283–e01214. doi: 10.1128/mBio.01283-14 25006228 PMC4161260

[B79] Pearce M. M. Zilliox M. J. Rosenfeld A. B. Thomas-White K. J. Richter H. E. Nager C. W.. (2015). The female urinary microbiome in urgency urinary incontinence. Am. J. Obstet. Gynecol. 213, 347 e1–347 11. doi: 10.1016/j.ajog.2015.07.009 PMC4556587 26210757

[B80] Philpot V. B. (1956). The bacterial flora of urine specimens from normal adults. J. Urol. 75, 562–568. doi: 10.1016/S0022-5347(17)66848-4 13296167

[B81] Pohl H. G. Groah S. L. Pérez-Losada M. Ljungberg I. Sprague B. M. Chandal N.. (2020). 'The urine microbiome of healthy men and women differs by urine collection method. Int. Neurourol. J. 24, 41–51. doi: 10.5213/inj.1938244.122 32252185 PMC7136448

[B82] Price T. K. Dune T. Hilt E. E. Thomas-White K. J. Kliethermes S. Brincat C.. (2016). The clinical urine culture: enhanced techniques improve detection of clinically relevant microorganisms. J. Clin. Microbiol. 54, 1216–1222. doi: 10.1128/JCM.00044-16 26962083 PMC4844725

[B83] Price T. K. Hilt E. E. Dune T. J. Mueller E. R. Wolfe A. J. Brubaker L. (2017). Urine trouble: should we think differently about UTI? Int. Urogynecol. J. 29, 205–210. doi: 10.1007/s00192-017-3528-8 29279968

[B84] Price T. K. Lin H. Gao X. Thomas-White K. J. Hilt E. E. Mueller E. R.. (2020). Bladder bacterial diversity differs in continent and incontinent women: a cross-sectional study. Am. J. Obstet. Gynecol. 223, 729.e1–29.e10. doi: 10.1016/j.ajog.2020.04.033 PMC7609606 32380174

[B85] Ravel J. Gajer P. Abdo Z. Schneider G. M. Koenig S. S. McCulle S. L.. (2011). Vaginal microbiome of reproductive-age women. Proc. Natl. Acad. Sci. U. S. A 108, 4680–4687. doi: 10.1073/pnas.1002611107 20534435 PMC3063603

[B86] Roberts W. (1881). On the occurrence of micro-organisms in fresh urine. Br. Med. J. 2, 623–625. doi: 10.1136/bmj.2.1085.623 PMC2263882 20750001

[B87] Roll-Hansen N. (1979). Experimental method and spontaneous generation: the controversy between Pasteur and Pouchet 1859–64. J. Hist. Med. Allied Sci. 34, 273–292. doi: 10.1093/jhmas/XXXIV.3.273 383780

[B88] Ronald L. S. Yakovenko O. Yazvenko N. Chattopadhyay S. Aprikian P. Thomas W. E.. (2008). Adaptive mutations in the signal peptide of the type 1 fimbrial adhesin of uropathogenic Escherichia coli. Proc. Natl. Acad. Sci. U. S. A 105, 10937–10942. doi: 10.1073/pnas.0803158105 18664574 PMC2504816

[B89] Ryan R. P. Dow J. M. (2008). Diffusible signals and interspecies communication in bacteria. Microbiol. (Reading). 154, 1845–1858. doi: 10.1099/mic.0.2008/017871-0 18599814

[B90] Saraf V. S. Sheikh S. A. Ahmad A. Gillevet P. M. Bokhari H. Javed S. (2021). Vaginal microbiome: normalcy vs dysbiosis. Arch. Microbiol. 203, 3793–3802. doi: 10.1007/s00203-021-02414-3 34120200

[B91] Schober I. Koblitz J. Sardà Carbasse J. Ebeling C. Schmidt M. L. Podstawka A.. (2025). Bac Dive in 2025: the core database for prokaryotic strain data. Nucleic Acids Res. 53, D748–DD56. doi: 10.1093/nar/gkae959 39470737 PMC11701647

[B92] Sedgley C. (2004). Root canal irrigation–a historical perspective. J. Hist. Dent. 52, 61–65. 15293717

[B93] Sfeir M. M. Hooton T. M. (2018). Practices of clinical microbiology laboratories in reporting voided urine culture results. Clin. Microbiol. Infect. 24, 669–670. doi: 10.1016/j.cmi.2017.12.023 29309935

[B94] Shafik A. Shafik I. El Sibai O. Shafik A. (2005). Changes in the urine composition during its passage through the ureter. A concept of urothelial function. Urol. Res. 33, 426–428. doi: 10.1007/s00240-005-0499-x 16284876

[B95] Short F. L. Murdoch S. L. Ryan R. P. (2014). Polybacterial human disease: the ills of social networking. Trends Microbiol. 22, 508–516. doi: 10.1016/j.tim.2014.05.007 24938173 PMC4158425

[B96] Siegman-Igra Y. (1994). Polymicrobial and monomicrobial bacteraemic urinary tract infection. J. Hosp. Infect. 28, 49–56. doi: 10.1016/0195-6701(94)90152-X 7806868

[B97] Siegman-Igra Y. Kulka T. Schwartz D. Konforti N. (1993). The significance of polymicrobial growth in urine: contamination or true infection. Scand. J. Infect. Dis. 25, 85–91. doi: 10.1080/00365549309169675 8460355

[B98] Siegman-Igra Y. Schwartz D. Konforti N. (1988). Polymicrobial bacteremia. Med. Microbiol. Immunol. 177, 169–179. doi: 10.1007/BF00232896 3393119

[B99] Siegman-IgraY (1994). The significance of urine culture with mixed flora. Curr. Opin. Nephrol. Hypertens. 3, 656–659. doi: 10.1097/00041552-199411000-00017 7881993

[B100] Simoni A. Schwartz L. Junquera G. Y. Ching C. B. Spencer J. D. (2024). Current and emerging strategies to curb antibiotic-resistant urinary tract infections. Nat. Rev. Urol. 21, 707–722. doi: 10.1038/s41585-024-00877-9 38714857 PMC11540872

[B101] Szczerbiec D. Piechocka J. Głowacki R. Torzewska A. (2022). Organic Acids Secreted by Lactobacillus spp. Isolated from Urine and Their Antimicrobial Activity against Uropathogenic Proteus mirabilis. Molecules 27, 5557. doi: 10.3390/molecules27175557 36080323 PMC9457960

[B102] Thomas-White K. Forster S. C. Kumar N. Van Kuiken M. Putonti C. Stares M. D.. (2018). Culturing of female bladder bacteria reveals an interconnected urogenital microbiota. Nat. Commun. 9, 1557. doi: 10.1038/s41467-018-03968-5 29674608 PMC5908796

[B103] Timm M. R. Russell S. K. Hultgren S. J. (2025). Urinary tract infections: pathogenesis, host susceptibility and emerging therapeutics. Nat. Rev. Microbiol. 23, 72–86. doi: 10.1038/s41579-024-01092-4 39251839 PMC13194463

[B104] Valenstein P. Meier F. (1998). Urine culture contamination: a College of American Pathologists Q-Probes study of contaminated urine cultures in 906 institutions. Arch. Pathol. Lab. Med. 122, 123–129. 9499354

[B105] Vollstedt A. Baunoch D. Wolfe A. Luke N. Wojno K. J. Cline K.. (2020). Bacterial interactions as detected by pooled antibiotic susceptibility testing (P-AST) in polymicrobial urine specimens. J. Surg. Urol. 1, 101. 36416755 PMC9678350

[B106] Wang D. Haley E. Luke N. Festa R. A. Zhao X Anderson L. A. Allison L. L.. (2023). Emerging and fastidious uropathogens were detected by M-PCR with similar prevalence and cell density in catheter and midstream voided urine indicating the importance of these microbes in causing UTIs. Infect. Drug Resist. 16, 7775–7795. doi: 10.2147/IDR.S429990 38148772 PMC10750486

[B107] Werneburg G. T. Hsieh M. H. (2024). Clinical microbiome testing for urology. Urol. Clin. North Am. 51, 493–504. doi: 10.1016/j.ucl.2024.06.007 39349017

[B108] Werneburg G. T. Lewis K. C. Vasavada S. P. Wood H. M. Goldman H. B. Shoskes D. A.. (2023). Urinalysis exhibits excellent predictive capacity for the absence of urinary tract infection. Urology 175, 101–106. doi: 10.1016/j.urology.2023.02.028 36898589

[B109] Werneburg G. T. Southgate J. (2024). Urinary microbiome research has not established a conclusive influence: revisiting koch's postulates. Eur. Urol. Focus 10, 686–687. doi: 10.1016/j.euf.2024.07.005 39089968

[B110] Whelan P. Nelson A. Kim C. J. Tabib C. Preminger G. Turner N. A.. (2022). Investigating risk factors for urine culture contamination in outpatient clinics: A new avenue for diagnostic stewardship. Antimicrob. Steward. Healthc. Epidemiol. 2, e29. doi: 10.1017/ash.2021.260 35445218 PMC9016366

[B111] Wise G. J. Schlegel P. N. (2015). Sterile pyuria. N. Engl. J. Med. 372, 1048–1054. doi: 10.1056/NEJMra1410052 25760357

[B112] Wojno K. J. Baunoch D. Luke N. Opel M. Korman H. Kelly C.. (2020). Multiplex PCR based urinary tract infection (UTI) analysis compared to traditional urine culture in identifying significant pathogens in symptomatic patients. Urol 136, 119–126. doi: 10.1016/j.urology.2019.10.018 31715272

[B113] Wolfe A. J. Toh E. Shibata N. Rong R. Kenton K. Fitzgerald M.. (2012). Evidence of uncultivated bacteria in the adult female bladder. J. Clin. Microbiol. 50, 1376–1383. doi: 10.1128/JCM.05852-11 22278835 PMC3318548

[B114] Xu R. Deebel N. Casals R. Dutta R. Mirzazadeh M. (2021). A new gold rush: A review of current and developing diagnostic tools for urinary tract infections. Diagnostics 11, 479. doi: 10.3390/diagnostics11030479 33803202 PMC7998255

[B115] Xu R. Ritts R. Badlani G. (2024). Unresolved pyuria. Curr. Bladder. Dysfunct. Rep. 19, 1–9. doi: 10.1007/s11884-023-00730-6

[B116] Zandbergen L. E. Halverson T. Brons J. K. Wolfe A. J. de Vos M. G. J. (2021). The good and the bad: ecological interaction measurements between the urinary microbiota and uropathogens. Front. Microbiol. 12, 659450. doi: 10.3389/fmicb.2021.659450 34040594 PMC8141646

[B117] Zering J. Stohs E. J. (2024). 'Urine polymerase chain reaction tests: stewardship helper or hinderance? Antimicrob. Steward. Healthc. Epidemiol. 6, e77. doi: 10.1017/ash.2024.71 PMC11077600 38721490

