# Supplementary material for: Polymicrobial urine cultures: reconciling contamination with the urobiome while recognizing the pathogens
Source: Front Cell Infect Microbiol. 2025 May 19;15:1562687. doi: 10.3389/fcimb.2025.1562687 (PMC12127331; doi:10.3389/fcimb.2025.1562687)
Supplement: Supplementary Table 1 — Most abundant skin microbes by region/nichea and incidence in urobiomeb. a From the top ten most abundant microbes in four different skin regions (Byrd et al., 2018). Note that some microbes are abundant in multiple regions (for ex., Corynebacterium tuberculostearicum and Staphylococcus epidermidis). Across four regions with ten microbes each, there are 22 unique taxa. b Microbes in bold type were found in the human female urobiome (Du et al., 2024) as determined by sequencing of genomes from isolates from catheterized urinary bladder urine. c Skin regions/niches are defined as follows: Dry: Hypothenar palm and volar forearm. Moist: Nare, antecubital fossa, inguinal crease, interdigital web, and popliteal fossa. Sebaceous: Alar crease, cheek, glabella, external auditory canal, manubrium, retroauricular crease, occiput, back. Feet:|Toe web space, toenail, andplantar heel. d Bacterial Diversity Database. See (Schober et al., 2025). e Also known as Cutibacterium acnes. [file DataSheet1.pdf]

Supplemental Materials

Polymicrobial Urine Cultures: Reconciling Contamination with the Urobiome while Recognizing the Pathogens

Robert B. Moreland[1], Linda Brubaker[2] and Alan J. Wolfe[1,*]

[1] Department of Microbiology and Immunology, Loyola University Chicago, Maywood, IL USA
[2] Department of Obstetrics, Gynecology and Reproductive Sciences, University of California San Diego, La Jolla, CA, USA.

* Correspondence: Alan J Wolfe, awolfe@luc.edu

SUPPLEMENTARY TABLE 1. MOST ABUNDANT SKIN MICROBES BY REGION/NICHE[a] AND INCIDENCE IN UROBIOME[b]

MICROBE	GRAM STAIN	OXYGEN TOLERANCE	REGION/NICHE[c]	TYPE STRAIN BACTERIAL DIVERSITY DATABASE[d]
Corynebacterium tuberculostearicum	Positive	Aerobe	Dry, Moist, Sebaceous, Feet	https://bacdive.dsmz.de/strain/3221
Staphylococcus epidermidis	Positive	Facultative Anaerobe	Dry, Moist, Sebaceous, Feet	https://bacdive.dsmz.de/strain/14529
Corynebacterium simulans	Positive	Facultative Anaerobe	Moist, Sebaceous, Feet	https://bacdive.dsmz.de/strain/3187
Micrococcus luteus	Positive	Aerobe/Obligate aerobe	Dry, Moist, Feet	https://bacdive.dsmz.de/strain/7673
Propionibacterium acne[e]	Positive	Anaerobe	Dry, Moist, Sebaceous	https://bacdive.dsmz.de/strain/133242
Staphylococcus capitis	Positive	Aerobe	Dry, Moist, Feet	https://bacdive.dsmz.de/strain/14602
Staphylococcus hominis	Positive	Aerobe	Moist, Sebaceous, Feet	https://bacdive.dsmz.de/strain/14636
Corynebacterium afermentans	Positive	Aerobe	Moist, Feet	https://bacdive.dsmz.de/strain/3167
Streptococcus mitis	Positive	Aerobe	Dry, Sebaceous	https://bacdive.dsmz.de/strain/14734
Corynebacterium amycolatum	Positive	Obligate Aerobe	Sebaceous	https://bacdive.dsmz.de/strain/3134
Corynebacterium aurimucosum	Positive	Facultative Anaerobe,	Sebaceous	https://bacdive.dsmz.de/strain/3199
Corynebacterium fastidiosum	Positive	Microaerophile	Moist	https://bacdive.dsmz.de/strain/135127
Corynebacterium kroppenstedtii	Positive	Facultative Anaerobe	Sebaceous	https://bacdive.dsmz.de/strain/3184
Corynebacterium resistens	Positive	Microaerophile	Foot	https://bacdive.dsmz.de/strain/3226
Enhydrobacter aerosaccus	Negative	Aerobe	Moist	https://bacdive.dsmz.de/strain/130456
Staphylococcus capitis	Positive	Obligate Aerobe	Sebaceous	https://bacdive.dsmz.de/strain/14506
Staphylococcus haemolyticus	Positive	Aerobe	Foot	https://bacdive.dsmz.de/strain/14544
Staphylococcus warneri	Positive	Aerobe	Foot	https://bacdive.dsmz.de/strain/14587
Streptococcus oralis	Positive	Microaerophile	Dry	https://bacdive.dsmz.de/strain/14740
Streptococcus pseudopneumonia	Positive	Microaerophile	Dry	https://bacdive.dsmz.de/strain/14831
Streptococcus sanguinis	Positive	Microaerophile	Dry	https://bacdive.dsmz.de/strain/14767
Veillonella parvula	Negative	Anaerobe	Dry	https://bacdive.dsmz.de/strain/17169

NOTES

[a] From the top ten most abundant microbes in four different skin regions (Byrd et al., 2018). Note that some microbes are abundant in multiple regions (for ex., *Corynebacterium tuberculostearicum* and *Staphylococcus epidermidis*). Across four regions with ten microbes each, there are 22 unique taxa.
[b] Microbes in bold type were found in the human female urobiome (Du et al., 2024) as determined by sequencing of genomes from isolates from catheterized urinary bladder urine.
[c] Skin regions/niches are defined as follows: Dry: Hypothenar palm and volar forearm. Moist: Nare, antecubital fossa, inguinal crease, interdigital web, and popliteal fossa. Sebaceous: Alar crease, cheek, glabella, external auditory canal, manubrium, retroauricular crease, occiput, back. Feet:|Toe web space, toenail, andplantar heel.
[d] For Bacterial Diversity Database , see (Schober et al., 2025)
[e] Also known as *Cutibacterium acnes*

SUPPLEMENTARY TABLE 2. MOST ABUNDANT VAGINAL MICROBES[a] AND INCIDENCE IN UROBIOME[b]

MICROBE	GRAM STAIN	OXYGEN TOLERANCE	COMMUNITY GROUP/CONDITION[c]	TYPE STRAIN BACTERIAL DIVERSITY DATABASE[d]
Lactobacillus crispatus	Positive	Anaerobe	Community Group I dominant	https://bacdive.dsmz.de/strain/6442
Lactobacillus gasseri	Positive	Anaerobe	Community Group II dominant	https://bacdive.dsmz.de/strain/6466
Lactobacillus jensenii	Positive	Anaerobe	Community Group V dominant	https://bacdive.dsmz.de/strain/6475
Lactobacillus iners	Positive	Anaerobe	Community Group III dominant	https://bacdive.dsmz.de/strain/6580
Gardnerella vaginalis	Variable	Anaerobe	Community Group IV, Bacterial vaginosis[c]	https://bacdive.dsmz.de/strain/1757
Atopobium vaginae[e]	Positive	Anaerobe	Community Group IV, Bacterial vaginosis[c]	https://bacdive.dsmz.de/strain/3043
Prevotella bivia	Negative	Anaerobe	Community Group IV, Bacterial vaginosis[c]	https://bacdive.dsmz.de/strain/12525

NOTES

[a] Ravel et al 2011 evaluated vaginal microflora and vaginal pH in 396 asymptomatic sexually active women representing four ethnic groups (white, black, Hispanic, and Asian) by pyrosequencing of 16S rRNA genes. *Lactobacillus* species dominated four groups, but one group accounting for 27% of women (termed as group IV community state type) lacked significant lactobacilli and was heterogeneous with higher proportion of strictly anaerobic bacteria including genera *Gardnerella*, *Prevotella*, *Atopobium*, as well as *Dialister*, *Megasphaera*, *Peptoniphilus*, *Sneathia*, *Eggerthella*, *Aerococcus*, *Finegoldia*, and *Mobiluncus*. This latter group was over-represented in black and Hispanic women (Ravel et al., 2011).
[b] Microbes in bold type were found in the human female urobiome (Du et al., 2024) as determined by sequencing of genomes from isolates from catheterized urinary bladder urine.
[c] For bacterial vaginosis, see (Saraf et al., 2021)
[d] For Bacterial Diversity Database , see (Schober et al., 2025)
[e] *Atopobium vaginae* is now known as *Fannyhessea vaginae* (Nouioui et al., 2018)

LITERATURE CITED

Byrd AL, Belkaid Y., Segre JA (2018). "The human skin microbiome." Nat Rev Microbiol **16**(3): 143-155.
Du J, Khemmani M., Halverson T, Ene A, Limeira R, Tinawi L, Hochstedler-Kramer BR, Noronha MF, Putonti C, Wolfe AJ (2024). "Cataloging the phylogenetic diversity of human bladder bacterial isolates." Genome Biol **25**(1): 75.
Nouioui I, Carro L., García-López M, Meier-Kolthoff JP, Woyke T, Kyrpides NC, Pukall R, Klenk HP, Goodfellow M, Göker M (2018). "Genome-Based Taxonomic Classification of the Phylum Actinobacteria." Front Microbiol **9**: 2007.
Ravel J, Gajer P., Abdo Z, Schneider GM, Koenig SS, McCulle SL, Karlebach S, Gorle R, Russell J, Tacket CO, Brotman RM, Davis CC, Ault K, Peralta L, Forney LJ (2011). "Vaginal microbiome of reproductive-age women." Proc Natl Acad Sci U S A **108**: 4680-4687.
Saraf VS, Sheikh S., Ahmad A, Gillevet PM, Bokhari H, Javed S (2021). "Vaginal microbiome: normalcy vs dysbiosis." Arch Microbiol **203**(7): 3793-3802.
Schober I, K. J., Sardà Carbasse J, Ebeling C, Schmidt ML, Podstawka A, Gupta R, Ilangovan V, Chamanara J, Overmann J, Reimer LC (2025). "Bac Dive in 2025: the core database for prokaryotic strain data." Nucleic Acids Research **53**(D1): D748–D756.
